# *Autographivirinae* Bacteriophage Arno 160 Infects *Pectobacterium carotovorum* via Depolymerization of the Bacterial O-Polysaccharide

**DOI:** 10.3390/ijms21093170

**Published:** 2020-04-30

**Authors:** Mikhail M. Shneider, Anna A. Lukianova, Peter V. Evseev, Anna M. Shpirt, Marsel R. Kabilov, Anna D. Tokmakova, Kirill K. Miroshnikov, Ekaterina A. Obraztsova, Olga A. Baturina, Alexander S. Shashkov, Alexander N. Ignatov, Yuriy A. Knirel, Konstantin A. Miroshnikov

**Affiliations:** 1 Shemyakin-Ovchinnikov Institute of Bioorganic Chemistry, Russian Academy of Sciences, 117997 Moscow, Russia; mm_shn@mail.ru (M.M.S.); a.al.lukianova@gmail.com (A.A.L.); petevseev@gmail.com (P.V.E.); anna.zem@mail.ru (A.D.T.); infon18@gmail.com (K.K.M.); e.a.obraztsova@gmail.com (E.A.O.); 2 Department of Biology, Lomonosov Moscow State University, 119991 Moscow, Russia; 3 Zelinsky Institute of Organic Chemistry, Russian Academy of Sciences, 119991 Moscow, Russia; asyashpirt@gmail.com (A.M.S.); alexander.shashkov@mail.ru (A.S.S.); yknirel@gmail.com (Y.A.K.); 4 Institute of Chemical Biology and Fundamental Medicine, Siberian Branch of Russian Academy of Sciences, 630090 Novosibirsk, Russia; kabilov@niboch.nsc.ru (M.R.K.); baturina@niboch.nsc.ru (O.A.B.); 5 Skryabin Moscow State Academy of Veterinary Medicine and Biotechnology, 109377 Moscow, Russia; 6 Winogradsky Institute of Microbiology, Federal Research Center “Fundamentals of biotechnology”, Russian Academy of Sciences, 117312 Moscow, Russia; 7 Research Center “PhytoEngineering” Ltd., Rogachevo, 141880 Moscow, Russia; an.ignatov@gmail.com

**Keywords:** *Pectobacterium carotovorum*, bacteriophage, taxonomy, *Autographivirinae*, O-specific polysaccharide, lipopolysaccharide, random sugar O-acetylation, tail spike protein, rhamnosidase

## Abstract

Phytopathogenic bacteria belonging to the *Pectobacterium* and *Dickeya* genera (soft-rot *Pectobacteriaceae*) are in the focus of agriculture-related microbiology because of their diversity, their substantial negative impact on the production of potatoes and vegetables, and the prospects of bacteriophage applications for disease control. Because of numerous amendments in the taxonomy of *P. carotovorum*, there are still a few studied sequenced strains among this species. The present work reports on the isolation and characterization of the phage infectious to the type strain of *P. carotovorum*. The phage Arno 160 is a lytic Podovirus representing a potential new genus of the subfamily *Autographivirinae*. It recognizes O-polysaccahride of the host strain and depolymerizes it in the process of infection using a rhamnosidase hydrolytic mechanism. Despite the narrow host range of this phage, it is suitable for phage control application.

## 1. Introduction

Soft rot *Pectobacteriaceae* (SRP) causing soft rot and black leg in potatoes and vegetables are worldwide phytopathogens that pose a serious threat to agriculture [1,2]. In recent years, the taxonomy of *Pectobacterium* and *Dickeya* genera comprising SRP have undergone numerous refinements and redistributions. Compared to the 1990s, when most SRP were considered to be representatives of the *Erwinia* genus (and some phytopathologists still use the term “pectolytic *Erwinia*” due to the similarity of symptoms), the current taxonomic situation with SRP is that there are almost 30 species, defined according to the plant host, severity of the resulting disease, adaptation to particular environmental conditions and, mostly, genomic features [3,4,5]. Most taxonomic alterations have been applied to the species *Pectobacterium carotovorum* (Pca), formerly *Erwinia carotovora*. Several species have been separated from Pca [6,7,8,9], and several subspecies have been elevated to species level [10]. Therefore, the number of bacterial strains still attributed as *Pectobacterium carotovorum* with genomic data deposited to the NCBI GenBank is now limited. 

Currently, several applications employing specific bacterial viruses (bacteriophages) are offered as a valid method to prevent and treat bacterial infections and, thus, minimize crop loss. To provide a scientific basis to the use of phage control, precise diagnostics of the pathogen is required, due to the high specificity of most known bacteriophages [11]. Moreover, it is difficult to assess whether the bacteriophages previously described as phages infectious to Pca are indeed infectious for this host. Unlike *Pectobacterium atrosepticum* phages, where the genomic diversity within the species is not high, and the sequenced type strains have been used for phage isolation and propagation [12,13], most phages of Pca described in publications have been isolated using poorly characterized field isolates [14,15,16]. The goal of the presented work was to isolate and characterize the phage infective to the type strain referred to as Pca, and to find out the mechanism of host recognition based on the interaction of tail spike protein with O-polysaccharide on the surface of the bacterium.

## 2. Results

### 2.1. Bacteriophage Arno 160 

#### 2.1.1. Biology and Morphology

**Figure 1:**
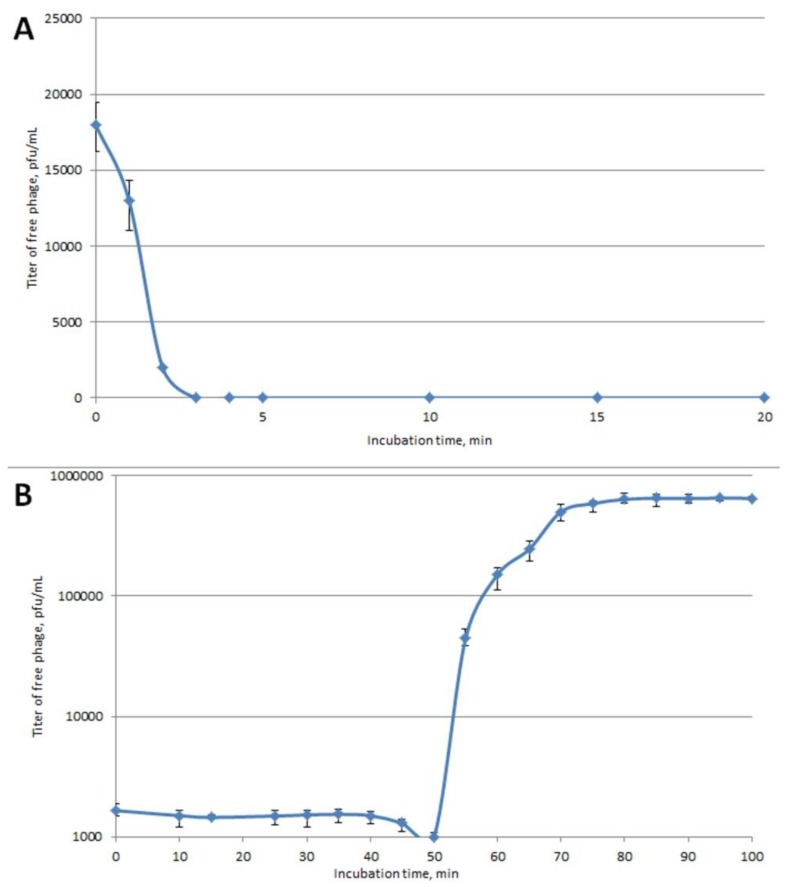
(**A**) Adsorption of phage Arno 160 to host bacteria. (**B**) One step growth curve of Arno 160 using *P. carotovorum* F160 as a host.

**Figure 2:**
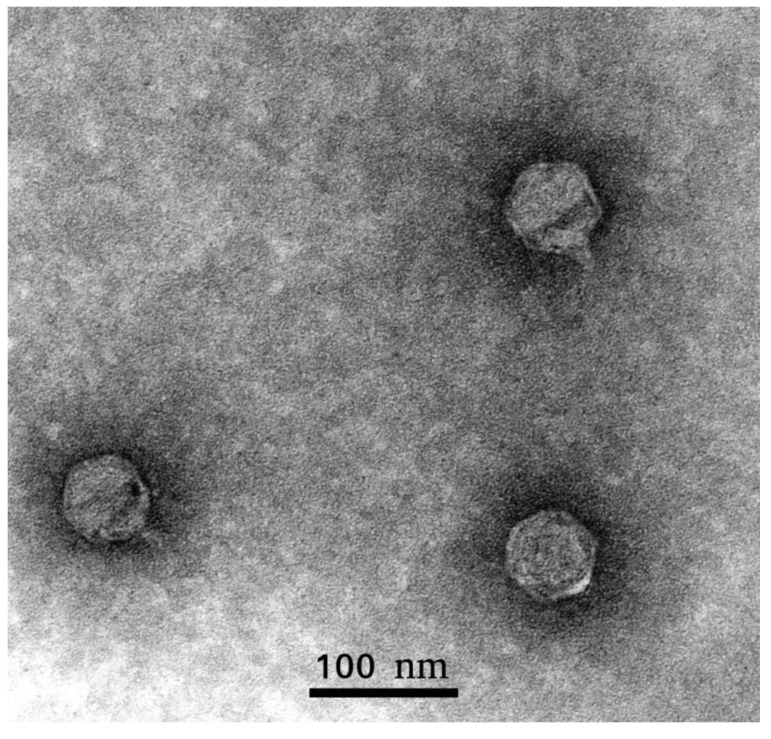
Electron micrograph of Arno 160 virions. Specimens were contrasted with 1% uranyl acetate.

Phage Arno 160 was isolated in 2018, from a sample of river water from Arno, Italy. The type strain of *Pectobacterium carotovorum* F160 = VKM 1247 = ATCC 15,713 = DSM 30,168 = LMG 5702 = ICMP 5702 = NCPPB 312^T^ was used as a bacterial host. The phage possesses a narrow host range, in that it is only capable of forming plaques on the phage’s host strain (F160) and only one additional strain, F167, a filed isolate of 2018 attributed as Pca by PCR diagnostics (Supplementary Table S1). All other tested SRP strains belonging to *P. atrosepticum*, *P. parmentieri*, *P. versatile*, *P. aquaticum*, *Dickeya* spp. and non-pathogenic soil *Enterobacteriaceae*, usually accompanying the soft rot infection of potatoes, have been resistant to phage Arno 160. On the propagation host F160, the phage produces clear plaques with a ~3 mm diameter. Infectivity assays under standard conditions show fast adsorption of the phage, followed by a rather long 50-min latent period and a burst size of approximately 70 pfu/cell (Figure 1). The morphology of phage Arno 160, as shown by transmission electron microscopy (Figure 2), can be classified as *Podoviridae* morphotype C1 [17] with an icosahedral head (61 ± 4 nm in diameter) and a short, non-contractile tail (12 ± 3 nm) without distinguishable appendices. In accordance with the formal nomenclature [18], the phage should thus be named vB_PcaP_Arno 160.

#### 2.1.2. Genome Analysis

**Figure 3:**
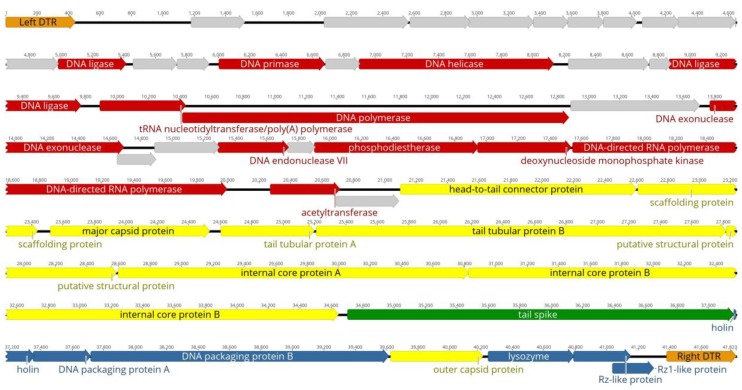
Genetic map of phage Arno 160. Nucleic acid metabolism, replication and transcription genes are colored in red, structural genes in yellow, adsorption apparatus genes in green, lysis genes in blue. Hypothetical protein genes are colored in grey.

The genome of Pca phage Arno 160 (MK053931) sequenced with a 4337× coverage is a double-stranded DNA with a size of 41,823 bp (including 443-bp long terminal repeats) and average G + C content of 51.4%. The genome contains 48 putative ORFs oriented in the same direction on the same DNA strand (Figure 3). Such genome architecture is typical for *Autographivirinae* phages. Putative functions of 28 proteins can be predicted, and 20 ORFs are assigned as hypothetical proteins (Supplementary Table S2). No genes related to the lysogenic cycle were identified, so it is possible to consider the infection cycle of Arno 160 as lytic. Also, no genes of rRNA, tRNA, bacterial toxins, antibiotic resistance or virulence factors were found.

#### 2.1.3. Taxonomy

A search for evolutionary relatives of phage Arno 160 was conducted by average nucleotide identity (ANI) measurements using 2212 *Podoviridae* genomes in the NCBI GenBank database, and OrthoANIu (Supplementary Table S3). The results of the search pointed to *Pectobacterium* phage PP2 [19] as the closest possible relative of phage Arno 160. ANIb, ANIm and tetranucleotide usage [20] estimated with Jspecies (Supplementary Table S4) supported the close evolutionary relations between *Pectobacterium* phage PP2 and *Pectobacterium* phage Arno 160 genomes. Phage PP2 demonstrates 92.6% ANI identity with Arno 160, therefore these phages can be considered as separate species.

**Figure 4:**
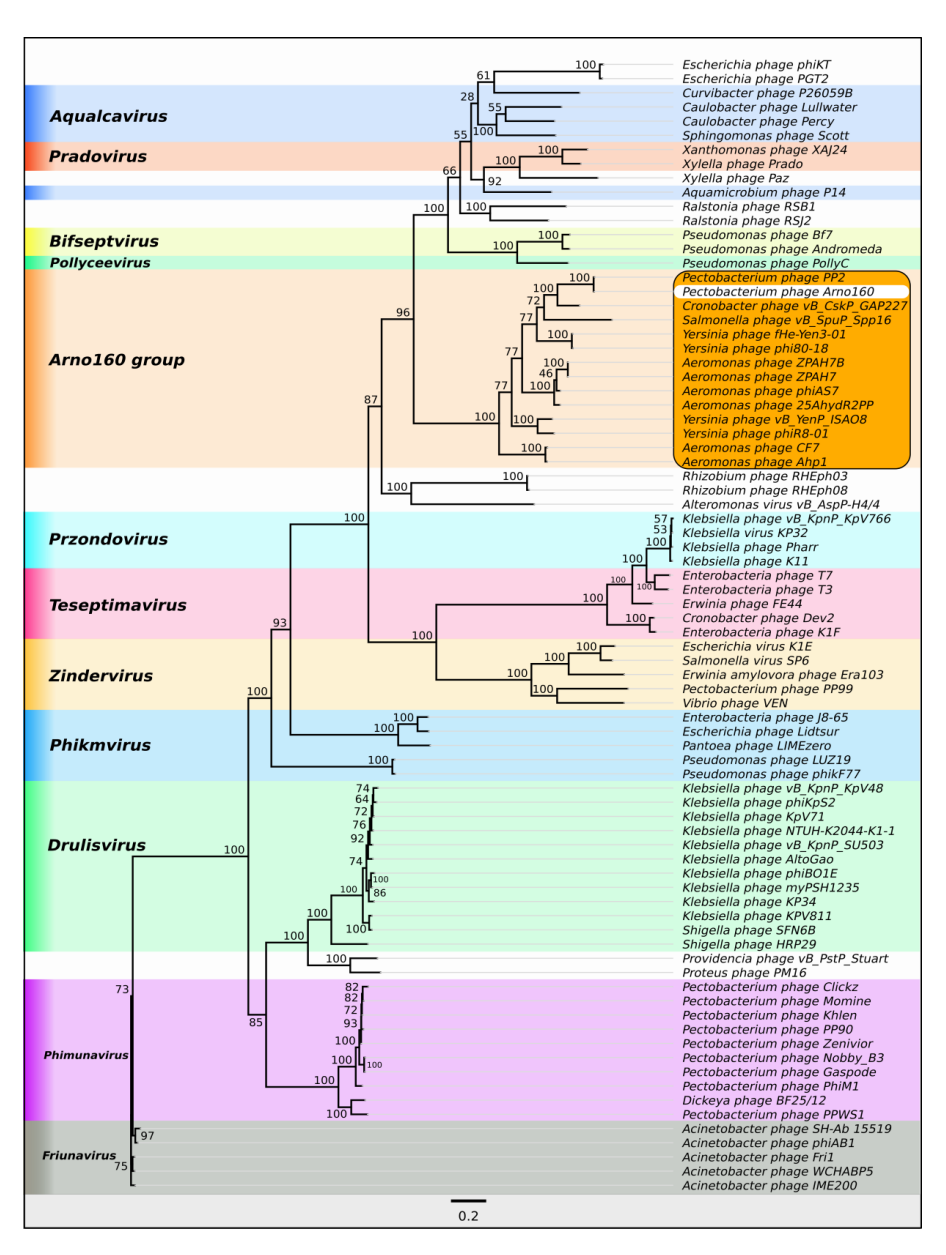
Phylogenetic tree of 80 concatenated amino sequences of phage DNA polymerase I, major capsid protein, DNA-directed RNA polymerase, head-to-tail connector protein and a large subunit of terminase (RAxML,GAMMA I BLOSSUM62 protein model, with 1000 bootstrap replicates).

**Figure 5:**
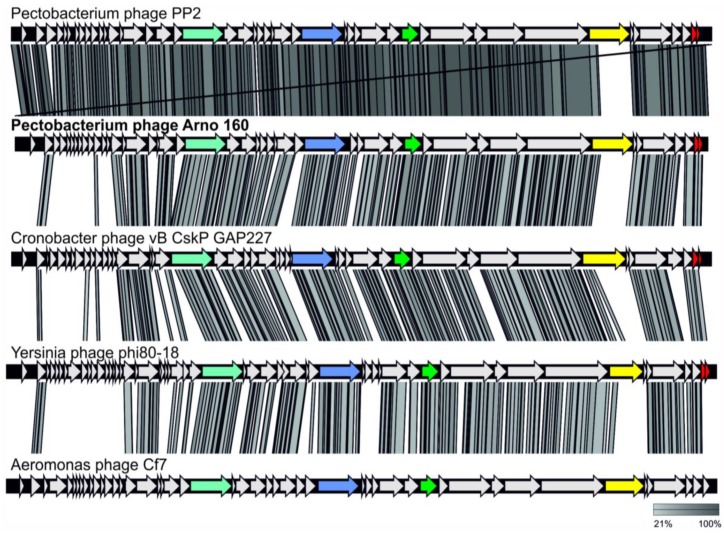
Pairwise comparison of the phage genomes *Pectobacterium* phage Arno 160, *Pectobacterium* phage PP2, *Cronobacter* phage vB_CskP_GAP227, *Yersinia* phage phi80–18 and *Aeromonas* phage CF7. Genomic maps were created using currently available annotation from Genbank with comparisons employing TBLASTX and visualization with Easyfig. DNA-polymerase genes are colored in cyan, RNA-polymerase genes are colored in blue, major capsid protein genes are colored in green, tail spike protein genes are colored in yellow and Rz/Rz1-like protein genes are colored in red.

As shown earlier, based on phylogenetic analysis, *Pectobacterium* phage PP2 and its homologous *Cronobacter* bacteriophages vB_CskP_GAP227 [21] and Dev-CD-23823 form an unclassified group within the subfamily *Autographivirinae* [19]. To clarify the evolutionary origin of Arno 160 and to find other members of this group, we conducted phylogenetic studies of amino acid sequences of five conservative genes (DNA polymerase I, major capsid protein, DNA-directed RNA polymerase, head-to-tail connector protein and a large subunit of terminase (DNA maturase B)) and their concatenated alignments. We extracted gene sequences from 80 phage genomes, including phage genomes found by ANI measurement and Arno 160 open-reading frames BLAST search using the custom Genbank phage genome database, and generated phylogenetic trees for translated sequences for each gene (Supplementary Figures S1–S5). All these trees confidently group *Pectobacterium* phage Arno 160 together with *Pectobacterium* phage PP2 and 12 other unclassified phages in a distinct monophyletic branch. We also generated a phylogenetic tree with concatenated alignments of these five conservative genes (Figure 4). The concatenated tree, along with the gene trees, supports the placement of the Arno 160 group in a clade clearly different from *Phimunavirus* and *Drulisvirus,* and other classified *Autographivirinae.* The bootstrap analysis supported the whole branch with 100% robustness. Heat map analysis (Supplementary Figure S6) supported the phylogeny results placing 14 unclassified phages in one group. Dot plot analysis demonstrated that Arno 160 represents a separate group of bacteriophages potentially ranked as a genus belonging to the *Autographivirinae* subfamily. These conclusions are in agreement with the proposals in [19,21]. Comparative analysis of the genomes of the taxonomic group including Arno 160 (Figure 5) showed a conservative gene order. The similar gene order could reflect the close principles of gene regulation in the genomes of this phage group.

#### 2.1.4. Adsorption Proteins

**Figure 6:**
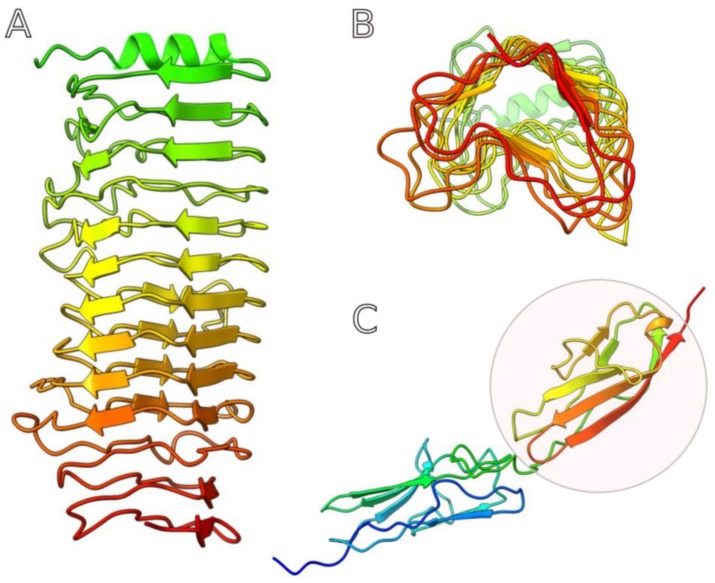
Homology modelling of the *phage* Arno 160 tail spike central region (**A**,**B**) and outer capsid protein (**C**), performed with best-fitting HHpred templates (toolkit.tuebingen.mpg.de). The tail spike contains parallel β-helices organized as triangular β-prisms (**B**), and the outer capsid protein contains an Ig-like domain (circled). The models are colored based on rainbow gradient scheme where the N-terminus of the polypeptide chain is colored blue, and the C-terminus is colored red.

The adsorption apparatus of Arno 160 consists of tail spikes formed by a single protein encoded by ORF 41. HHpred PDB search predicted the structure enriched with parallel beta strands, similar to the tail spike protein (TSP) of *Acinetobacter* bacteriophage ΦAB6 [22]. Comparative protein structure modelling of the Arno 160 tail spike predicted a central β-sheet region that forms a right-handed, parallel β-helix with triangular β-prisms (Figure 6). This region could form the receptor-binding domain, as well as a similar central domain in the ΦAB6 tail spike [22]. It was shown that ΦAB6 TSP specifically degraded the lipopolysaccharide of the bacterial host strain, supposedly the O-antigen side chain [22]. Thus, we can propose the action of the Arno 160 TSP in a similar way and the presence of a polysaccharide-depolymerizing domain in gp41. The N-terminal part of TSP (aa 13–133) contains the predicted domain PF03906.14 (phage_T7_tail protein) followed by a long a-helical domain (aa 100–250). We propose this part of the protein to be responsible for the attachment of the TSP to the virion.

Recombinant TSP Arno 160 gp41, lacking the N-terminal part (aa 1–217), can be produced in the *E. coli* expression system in biologically active form using the SlyD-fusion strategy. N-terminal chimeric attachment of *E. coli* SlyD, a peptidyl-prolyl isomerase, was previously shown to enhance expression and to stabilize recombinant fibrous proteins [23]. After proteolytic removal of the SlyD folding driver, recombinant gp41 retains a stable trimeric form, and can be purified to electrophoretic homogeneity. The protein is stable for several weeks at 4 °C.

We also suggest the possible participation of outer capsid protein (OCP) encoded by ORF45 in some kind of phage adsorption (Figure 6). Protein database searches (InterProScan and PDB with HHpred) predicted the presence of an Ig-like domain in the C-terminal part of OCP. Ig-like domains are found commonly in structural proteins of tailed dsDNA phages [24]. There are several hypotheses proposing the role of capsid proteins containing an Ig-like domain [24]. T4-like bacteriophage RB49 Hoc protein (highly antigenic outer capsid protein) decorating the outer surface of a capsid contains three domains with an immunoglobulin-like fold, and, as has been shown by biochemical experiments, can bind to *E. coli* cells [25]. It has also been supposed that Hoc might allow the phage to use a bacterium (which is not necessarily its host) as a “vehicle” for travel to different locations [25]. Meanwhile, an Ig-like domain-containing decoration protein pb10 of phage T5 may function to reinforce the capsid, thus favoring phage survival in harsh environments [26]. The orthologs of Arno 160 ORF45 are conserved within the genomes of phages comprising the putative genus. However, the functions of capsid decoration proteins of *Autographivirinae* phages have not been studied in detail previously, so we are unable to indicate the role of gp45. 

#### 2.1.5. Lysis Cassette

Previous papers describing phages similar to Arno 160, *Pectobacterium* phage PP2 [19] and *Cronobacter* phage vB_CskP_GAP227 [21] indicate the presence of a dual host lysis system involving lysins and holins. The genome of Arno 160 contains genes encoding the peptidoglycan-lysing enzyme, which has been proposed to be muramidase (ORF46), and the distantly located holin (ORF42). However, the search also revealed the presence of two small ORFs, 47 and 48, with high similarity to known Rz-like and Rz1-like proteins, respectively. Such proteins have been shown to form a complex spanning the periplasmic space, providing more efficient lysis of the host cell [27]. Alignment by MAFFT shows the presence of genes encoding homologous proteins (pairwise identity > 96 for PP2) in almost the same positions in the genomes of phages PP2, vB_CskP_GAP227 and *Yersinia* phage phi80–18. Therefore, we propose that the phages of the group including Arno 160 encode the combined lysis module involving lysin, holin, and Rz-like/Rz1-like, proteins.

### 2.2. Pectobacterium Carotovorum Strain F160. Genomic Analysis

Phage Arno 160 is selected and propagated on a *Pectobacterium carotovorum* (previously *P. carotovorum* subsp. *carotovorum,* syn: *Erwinia carotovora*) type strain. This strain, F160 = VKM 1247 = ATCC 15,713 = DSM 30,168 = LMG 5702 = CFBP 2046 = ICMP 5702 = NCPPB 312^T^, was initially isolated in Denmark in the 1960s, and is used in international collections of microorganisms as a model representative of the species [5]. Draft genomes of this strain are available at NCBI GenBank as ICMP 5702 (NZ_AODT00000000.1) [28], NCPPB 312 (NZ_JQHJ00000000.1, directly submitted in 2014) and DSM30168 (NZ_FQWI00000000.1, directly submitted in 2016). These sequences are identical, except for gaps between contigs, and a few nucleotide polymorphisms. 

**Figure 7:**
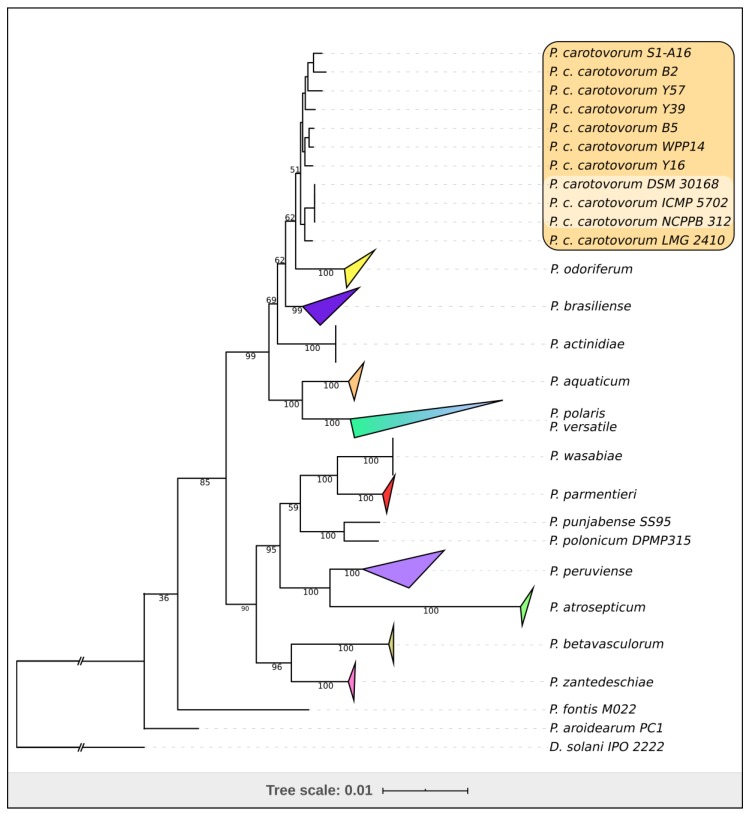
Phylogenetic tree of 147 concatenated amino sequences of 51 ribosomal proteins (RAxML,GAMMA I BLOSSUM62 protein model, with 1000 bootstrap replicates). Eleven *Pectobacterium* strains highlighted in bold form a monophyletic clade including the strain F160 (*P. carotovorum* NCPPB 312 = ICMP 5702 = DSM 30,168 type strain). *Dickeya solani* IPO 2222 was used as an outgroup. The expanded branches are shown in Supplemental Figure S7.

We generated a phylogenetic tree with concatenated alignments of 51 ribosomal proteins of 147 *Pectobacterium* strains (Figure 7, Supplemental Figure S7). The tree supports a placement of another eight strains deposited as *P. c.* subsp. *carotovorum* to the monophyletic clade, together with three sequences of the type strain. These strains are: LMG 2410 (NZ_VBUA00000000.1), WPP14 (NZ_ABVY00000000.1) [29], B2 (NZ_JUJR00000000.1), B5 (NZ_JUJS00000000.1), Y16 (NZ_JUJO00000000.1), Y39 (NZ_JUJQ00000000.1), Y57 (NZ_JUJG00000000.1) and S1-A16 (NZ_OZDJ00000000.1). The clade comprises strains isolated in a broad range of geographic locations (USA, Great Britain, China and Morocco) and different host plants (potato, cucumber, Chinese cabbage). This observation correlates with the definition of Pca as a worldwide multi-host phytopathogen [10,30,31].

### 2.3. Structure of the O-polysaccharide of P. carotovorum F160

**Figure 8:**
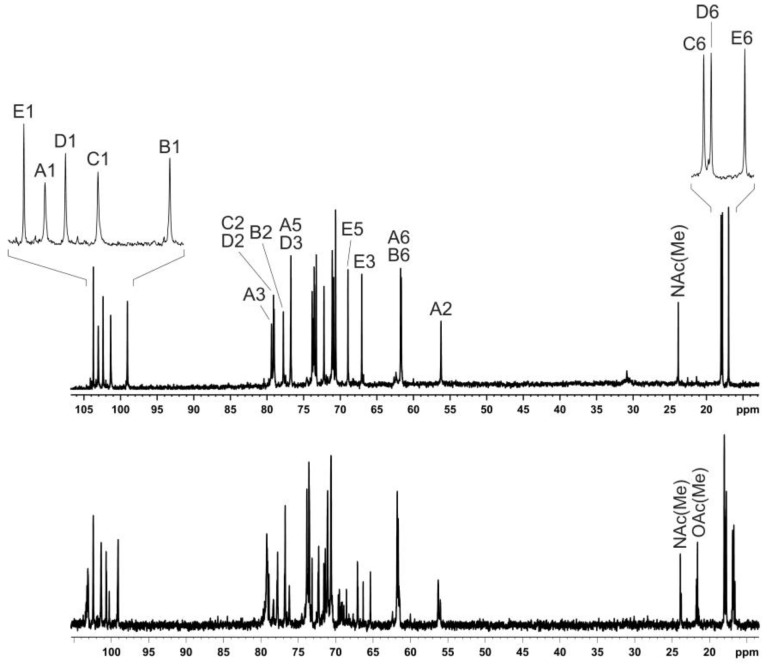
^13^C NMR spectra of the O-deacetylated polysaccharide (top) and O-polysaccharide (bottom). The region for CO groups is not shown. Numbers refer to carbons in sugar residue denoted by letters as indicated in Table 1 and Chart 1. *E1*, *E6* and E1, E6 indicate 6dTal residues that do, and do not, include the O-acetyl groups, respectively.

Sugar analysis of the OPS of Pca F160 revealed 6-deoxy-talose (6dTal), rhamnose, glucose and glucosamine in the ratios 1:1.5:1.8:1 (GLC detector response), respectively. The absolute configurations of the monosaccharides were established by analysis of ^13^C NMR data of the OPS taking into account known regularities in glycosylation effects [32], and the l configuration of rhamnose was confirmed by GLC of the peracetylated (*S*)-2-octyl rhamonosides [33]. The ^1^H NMR and ^13^C NMR (Figure 8, bottom) spectra showed significant structural heterogeneity of the OPS due to non-stoichiometric O-acetylation (there were multiple signals for O-acetyl groups at δ_H_ 2.15-2.24 and δ_C_ 21.5–21.8).

The OPS was O-deacetylated with aqueous ammonia to give a regular O-deacetylated polysaccharide (DPS). Its ^13^C NMR spectrum (Figure 8, top) contained, inter alia, signals for five anomeric carbons at δ 99.1–103.7, three *C*H_3_-C groups at δ 17.0, 17.8 and 18.0 (C-6 of Rha and 6dTal), two HO*C*H_2_-C groups at δ 61.7 and 61.8 (C-6 of Glc and GlcNAc), and one N-acetyl group at δ 23.8 (CH_3_) and 175.0 (CO). Accordingly, the ^1^H NMR spectrum of the DPS displayed signals for five anomeric protons at δ 4.68–5.47, three CH_3_-C groups at δ 1.26 (3H, d, *J*_5,6_ 5.9) and δ 1.31 (6H, d, *J*_5,6_ 6.1), and one N-acetyl group δ 1.95. These data demonstrated that the OPS had a pentasaccharide repeating unit containing two residues of l-Rha and one residue each of d-Glc, d-GlcNAc and l-6dTal. 

The ^1^H and ^13^C NMR spectra of the DPS were assigned (Table 1) using 1D ^1^H,^1^H TOCSY, 2D ^1^H,^1^H COSY, TOCSY, ROESY, ^1^H,^13^C HSQC and HSQC-TOCSY experiments. The pyranose forms of all monosaccharide residues and the configurations of the glycosidic linkages were established by ^13^C NMR chemical shifts of C-5 and compared with published data of the corresponding α- and β-pyranoses [34]. The β configuration of GlcNAc (unit **A**) was confirmed by a relatively large coupling constant *J*_1,2_ ~8 Hz and H-1/H-3 and H-1/H-5 correlations in the 2D ^1^H,^1^H ROESY spectrum. The α configuration of Glc (unit **B**) was affirmed by a H-1/H-2 correlation in the same spectrum.

Linkage and sequence analyses of the DPS were performed using the 2D ^1^H,^1^H ROESY spectrum, which displayed inter-residue correlations between the following anomeric protons and protons at the linkage carbons: GlcNAc **A** H-1/Rha **D** H-2, Glc **B** H-1/GlcNAc **A** H-3, Rha **C** H-1/Glc **B** H-2, Rha **D** H-1/Rha **C** H-2, 6dTal **E** H-1/Rha **D** H-3 at δ 4.68/4.18, 5.47/3.91, 5.24/3.65, 5.17/4.08 and 5.20/3.95. These findings were supported by ^1^H,^13^C HMBC and HSQC-NOESY experiments.

**Figure 9:**
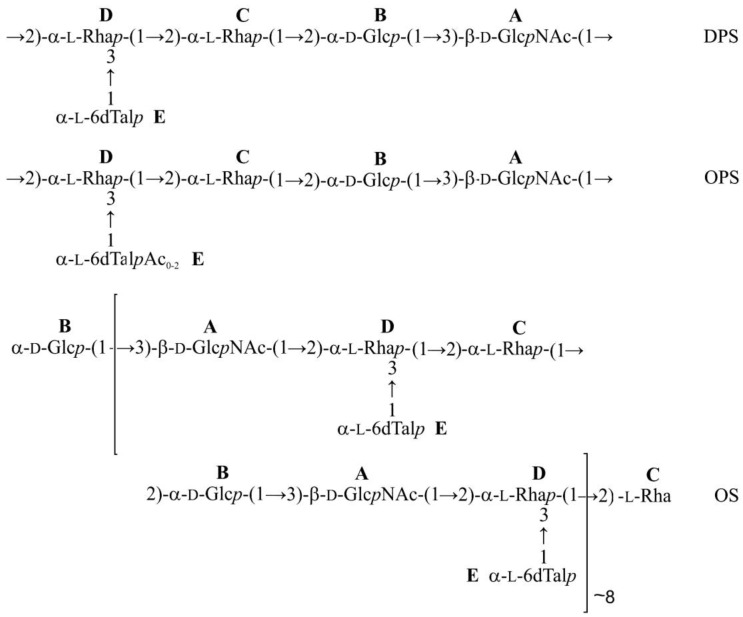
Structures of the O-deacetylated polysaccharide (DPS) (top), O-polysaccharide (OPS) (middle) from *Pectobacterium carotovorum* strain F160, and the oligosaccharide derived by depolymerization of the O-polysaccharide by tail spike protein gp41 (OS) (bottom).

The glycosylation pattern of the monosaccharides was confirmed by low-field positions of the linkage carbons, including C-3 of unit **A**, C-2 of units **B** and **C,** C-2 and C-3 of unit **D** at δ 76.7-79.4 in the ^13^C NMR spectrum of the DPS, as compared with their positions at δ 71.0-72.5 in the corresponding non-substituted monosaccharides [34,35]. The ^1^H and ^13^C NMR chemical shifts for 6dTal were similar to the published data for 6-deoxy-α-l-talose [35]. Based on the data obtained, it was concluded that the DPS had the structure shown in Figure 9. This structure is similar to that of *P. atrosepticum* SCRI 1039 [36], which differs only in the presence of a side-chain l-fucose residue in place of a 6-deoxy-l-talose residue. Analysis of published NMR spectroscopy data of the O-deacetylated polysaccharide from that bacterium showed that they are essentially identical to those of *P. carotovorum* F160 studied in this work. Particularly, the reported C-3 chemical shift of the 6-deoxy-α-hexose was δ 66.27 [36], which is similar to the value of δ 66.3 for 6-deoxy-α-l-talose [35] but significantly different from the C-3 chemical shift of α-fucose (δ 70.6 [37]).

**Figure 10:**
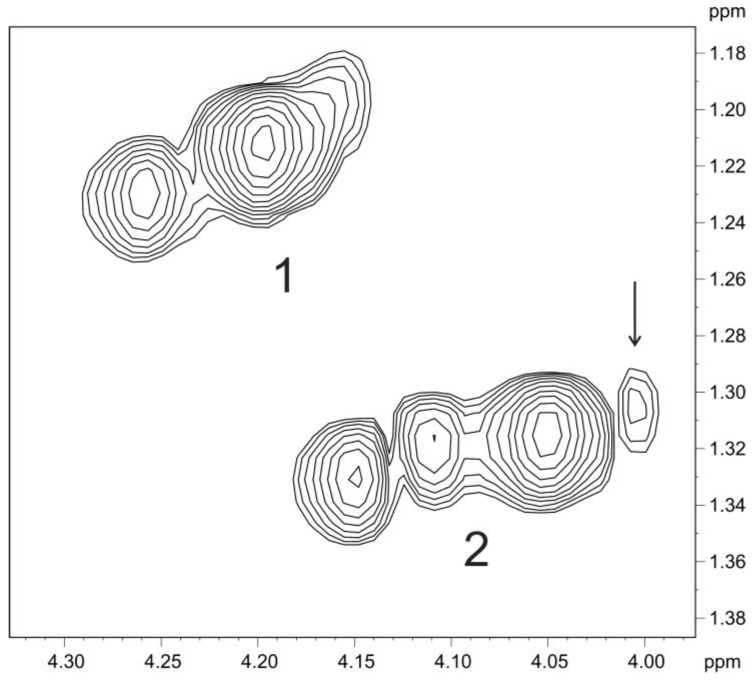
Part of a 600-MHz 2D ^1^H,^1^H COSY spectrum of the O-polysaccharide displaying 6dTal H-5/H-6 correlations. Two series of cross-peaks (1 and 2) were assigned to the O-acetylated forms of 6dTal that do, and do not, include the 4-*O*-acetyl group, respectively. The arrow points to the H-5/H-6 cross-peak of a nonacetylated 6dTal residue.

Comparison of the one- and two-dimensional NMR spectra of the initial OPS and DPS showed that the only O-acetylated monosaccharide in the OPS was 6dTal. This monosaccharide displayed multiple NMR signals, owing to the presence of various O-acetylated forms. Particularly, in the 2D ^1^H,^1^H COSY spectrum, there were seven H-5/H-6 cross-peaks for 6dTal, which formed two series 1 and 2 (Figure 10). Such an O-acetylation pattern is similar to that reported for the O-polysaccharide of *Aeromonas hydrophila* O:34 [38]. Series 2 of four cross-peaks contained the H-5/H-6 cross-peak for the nonacetylated form at δ 4.01/1.31, (in Figure 10, this cross-peak is indicated by an arrow, and may be compared with the 6dTal H-5/H-6 cross-peak at δ 4.01/1.31 in the COSY spectrum of the DPS). This cross-peak is minor, and hence most 6dTal residues in the OPS were O-acetylated. As the H-5 and H-6 chemical shifts are influenced mostly by an acetyl group at O-4, the three other peaks of series 2 were assigned to the O-acetylated forms that do not include the 4-*O*-acetyl group, including the 2-O-acetylated, 3-O-acetylated and 2,3-di-O-acetylated forms [37]. Correspondingly, the three cross-peaks of series 1 were assigned to the 4-O-acetylated, 3,4-di-O-acetylated and 2,4-di-O-acetylated forms of 6dTal. Therefore, the O-polysaccharide of *P. carotovorum* strain F160 has the structure shown in Figure 9 (middle).

### 2.4. Depolymerization of Bacterial Polysaccharide by the Tail Spike Protein gp41 

Both intact and O-deacetylated polysaccharides of *P. carotovorum* strain F160 were depolymerized by tail spike protein gp41. The product from the O-deacetylated polysaccharide was isolated by gel-permeation chromatography and studied by two-dimensional ^1^H,^1^H COSY and ^1^H,^13^C HSQC spectroscopy. This product was found to be a large oligosaccharide (OS) containing an average of eight pentasaccharide repeating units of the O-polysaccharide (Figure 9). NMR analysis showed that the reducing end of the OS was occupied by the rhamnose residue C, and the non-reducing end by the glucose residue B, (compare the positions of the H-1/C-1 cross-peak of the linked Rha C in the OPS and the reducing Rha C in the OS at 5.24/101.3 and 5.20/94.0, respectively), and the H-2/C-2 cross-peak of the 2-substituted glucose residue B in the OPS and terminal non-reducing Glc B in the OS at 3.65/77.8 and 3.53/73.0, respectively. These data showed that the tail spike protein gp41 cleaves the O-polysaccharide of *P. carotovorum* strain F160 by the hydrolytic mechanism, by the glycosidic linkage between the Rha C and Glc B residues and is therefore a specific rhamnosidase.

## 3. Discussion

Bacteriophages are considered prospective tools to manage bacterial diseases. Areas that are potentially interested in the use of phages as antibacterials include medicine, the veterinary sector, the food industry and plant science. In agriculture, phage cocktails have been successfully tested for the control of bacterial phytopathogens of many economically important plants (reviewed in [39,40]. The potato is one of the most staple food plants, and dramatic losses of vegetating plants and ware potatoes because of black leg and soft rot caused by bacteria of *Pectobacterium* and *Dickeya* genera increase the demand for the effective and ecologically friendly antibacterial treatment the phages can provide. However, the employment of phages has come across some difficulties and drawbacks, including the instability of phages to UV radiation, hard and irreproducible penetration to the vascular system of plants, and the fast evolution of phage-resistant mutants of bacteria. All of these problems can be solved, but a substantial scientific effort is necessary. Additional complications have been presented by the recent numerous rearrangements of the taxonomy of Pectobacteria [3,10,41]. After the elevation of *Pectobacterium carotovorum* subspecies *actinidiae*, *brasiliense*, *odoriferum* and *versatile* to the species level [10], and the separation of new species *P. fontis* [6], *P. polaris* [7], *P. aquaticum* [8] and *P. parvum* [9] from *P. carotovorum,* the number of strains that are still considered as the members of the current *P. carotovorum* is limited. The current situation also promotes refinements in the estimation of the abundance of SRP in the pathogenesis of potatoes, as well as the attribution of previously found bacteriophages that were considered to be infective to *P. carotovorum* subspecies. Considering the proposed unified naming of bacteriophages [18], like vB_PcaP_Arno 160, where the isolation host should be named, the resulting nomenclature of pectobacterial phages may be puzzling, because of both continuous renaming of the hosts, and incorrect attribution of the host strain. Therefore, we have chosen the historical type strain of *P. carotovorum* for phage isolation in order to assess the abundance of the phages specific to this strain in the environment, to investigate the distribution of the strains susceptible to the isolated phage in potato soft rot pathogenesis in Russia, and to reveal the mechanism of phage recognition of the particular bacterial host. Several attempts to isolate the phage specific to the strain F160 from environmental samples taken in Russia have failed. Furthermore, the phage was previously isolated from a sample of river water from Italy, a country differing both in climate conditions, and the structure of agricultural plants. 

The isolated phage Arno 160 belongs to the family *Podoviridae*, of subfamily *Autographivirinae*. Lytic representatives of this subfamily are widespread in nature. Phage Arno 160 demonstrates an efficient lysis of infected bacteria (Figure 1) and, thus, as for most such phages, can be considered as being a suitable candidate for phage therapy applications. The host range of phage Arno 160 is narrow, covering the only characterized strain among the ample collection of most known species of *Pectobacterium* and *Dickeya* (Supplementary Table S1). Only one uncharacterized isolate of Pectobacteria circulating in the soft-rot pathogenesis in central Russia in 2017–2018 was susceptible to Arno 160. This means that strains directly related to the type strain of Pca (earlier Pcc) are seldom among current pathogens in Eastern Europe. However, due to the pronounced lytic activity of Arno 160, it can be considered to be among the therapeutic phages that might potentially be used in case of the evolution of Pca derivatives highly virulent to potatoes or other plants.

Accumulated information on the genomes of *Autographivirinae* phages has promoted the taxonomic division of this subfamily. Based on general genome sequence identity and the position/content of genes and gene cascades essential for the realization of the infection cycle, the phages of the *Autographivirinae* subfamily are currently distributed into nine genera and several unassigned species. The features of the phage Arno 160 genome attribute it as a representative of a new genus based on phylogenetic distances (Supplementary Figure S1–S5).

The adsorption apparatus of phage Arno 160 consists of tail spikes encoded by gene 41. Many *Podoviridae* phages tend to interact with bacterial polysaccharides (lipopolysaccharides, sugar moieties of the O-antigen or capsule polysaccharides) as primary receptors for host recognition [42]. Several phages infecting SRP have been experimentally shown to follow this rule [16,43,44,45]. Despite the diversity of species and strains comprising SRP, very limited information is available on both the composition of polysaccharides of *Pectobacterium* and *Dickeya*, and the details of phage interaction with the polysaccharides. Only eight structures of polysaccharides of *P. atrosepticum* [36,46,47], *P. wasabiae* [48], *P. carotovorum* (then *Erwinia carotovora* subsp. *carotovora*) [49], *P. brasilense* [45] and *D. solani* [44,50] have been identified. Thus, the research on pectobacterial polysaccharide is important for the development of phage therapy. In the present work, we have identified the structure of O-polysacchride of *P. carotovorum* type strain F160 = NCPPB 312^T^ (Figure 9). It differs from the OPS structure of the non-sequenced *P. carotovorum* strain GSPB 436 [49], and shows some similarity to the OPS of *P. atrosepticum* SCRI 1039 [36]. A noticeable feature of F160 OPS is a random acetylation of side-chain 6-deoxy-l-talose residues. 

The tail spike protein of Arno 160, gp 41 is a specific rhamnosidase, which degrades the OPS of the host strain using the hydrolytic mechanism. This degradation might allow the spatial access of the phage particle to the cell surface and subsequent injection of phage DNA inside the bacterium. Recombinant gp 41 splits the OPS into large fragments, above eight sugar units in size. If the native tail spike of the phage particle has the same enzymatic property, it may explain the relatively long lag period of the Arno 160 infection cycle (Figure 1). Understanding the principles of the interactions between the adsorption apparatus of the phage and the surface receptor of the bacterial host is beneficial for rational construction of phage combinations for therapeutic purposes.

## 4. Materials and Methods 

### 4.1. Bacterial Strains

The *Pectobacterium carotovorum* strain (*Pectobacterium carotovorum* subsp. *carotovorum* type strain (ATCC 15,713 = DSM 30,168 = LMG 5702 = ICMP 5702= NCPPB 312^T^) originated from the Russian collection of Microorganisms (VKM) and is designated as F160 in local Lab collection. Collection strains and field isolates of *Pectobacterium* and *Dickeya* spp. used for phage host range determination are shown in Supplementary Table S1. All strains were grown in Lysogeny Broth (LB) liquid media at intensive aeration or LB 1.5% agar at 28 °C.

### 4.2. Phage Isolation and Purification

Phage Arno 160 was isolated from the water of the river Arno (Pisa, Italy). *P. carotovorum* strain F160 was used for phage propagation. The phage was cultivated at 28 °C in LB using a standard protocol [51], with the titer in the resulting lysate of ~10^9^ PFU/mL. The phage was further purified by ultracentrifugation in CsCl gradient, dialyzed against PBS buffer and stored at 4 °C.

### 4.3. Electron Microscopy 

The morphology of phage Arno 160 was assessed by transmission electron microscopy. Purified phage suspension ~10^10^ PFU/mL was placed on individual copper grids, and then negatively stained with 1% uranyl acetate and examined using a Zeiss Libra 120 microscope at 100 kV acceleration voltage. The dimensions were averaged among ~20 individually measured particles.

### 4.4. Host Range and General Characterization

The infection range of Arno 160 was determined with a spot assay, as described previously [52]. Bacterial lawns of 40 strains representing different species and gene groups of SRP (Supplementary Table S1) on LB-agar plates were prepared by pouring 5 mL of soft LB agar (0.4% agar) inoculated with the bacterial culture. After solidification, 10 μL of serial dilutions of phage were applied, and dried for 20 min at room temperature. Plates were incubated overnight at 28 °C, and the phage plaques formed were counted the next day. The adsorption curve was plotted according to [51]. The host bacterial strain (Pca strain F160) was grown at 28 °C to an OD_600_ of ~0.25 (~1 × 10^8^ CFU/mL), then pelleted by centrifugation. The pellet was resuspended in phage suspension to yield an approximate multiplicity of infection (MOI) of 0.001, with subsequent incubation at 28 °C with moderate agitation. For adsorption assay 100 μL aliquots were taken at the indicated time points and transferred into 800 μL LB medium supplied with 50 μL chloroform. After bacterial lysis, the mixtures were centrifuged and the supernatant was titrated to determine the amount of non-adsorbed or reversibly adsorbed phages. For one-step-growth assays, an exponentially growing culture of host bacteria (10^7^ cfu/mL) was mixed with phage suspension (MOI of 0.01). The mixture was then incubated, with shaking, at 28 °C. At 10 min intervals, aliquots were taken to measure phage titer using the overlay method. All experiments were performed independently three to four times, and the results were averaged. 

### 4.5. Phage Sequencing and Annotation 

Phage DNA was fragmented with medium-size fragments of about 600 bp in a microTUBE Adaptive Focused Acoustics (AFA) fiber snap-cap tube using a Covaris S2 instrument (Covaris, Woburn, MA, USA). The DNA library was constructed using the dual-index NEBNext multiplex oligos (New England Biolabs, Ipswich, MA, USA) and the NEBNext Ultra II DNA library prep kit for Illumina (New England Biolabs). The library was size-selected on a Blue Pippin 1.5% agarose DNA gel (Sage Science, Beverly, MA, USA) with size-selection settings of 550–1000 bp. This DNA library was sequenced with reagent kit version 3 (600-cycle) on a MiSeq platform (Illumina) at the SB RAS Genomics Core Facility (ICBFM SB RAS, Novosibirsk, Russia). The entire genome was assembled de novo using SPAdes software version 3.11.1, with default parameters [53]. The phage genome was annotated by predicting and validating open reading frames (ORFs) using Prodigal 2.6.1 [54], GeneMarkS 4.3 [55] and Glimmer 3.02 [56]. Terminal repeats were identified using blasnt and by mapping the raw reads to the genome. Identified ORFs were manually curated to ensure fidelity. Functions were assigned to ORFs using a BLAST search on NCBI databases (http://blast.ncbi.nlm.nih.gov), InterProScan [57], HHpred (https://toolkit.tuebingen.mpg.de/#/tools/hhpred) [58], using databases PDB, SCOP, Pfam. NCBI_CONSERVED. tRNA coding regions were identified with tRNAscan-SE [59] and ARAGORN [60]. Resulting genomes were visualized using Geneious Prime, version 2020.0.3 (https://www.geneious.com). All annotated genes were compared against the Antibiotic Resistance Genes Database (ARDB, https://card.mcmaster.ca/) and the virulence factor database (VFDB, downloaded from http://www.mgc.ac.cn/VFs/). The annotated genome of phage Arno 160 has been deposited in the NCBI GenBank under Accession number MK053931.

### 4.6. Phylogeny and Taxonomy Studies

Bacterial and phage reference genomes were downloaded from the NCBI Genbank (ftp://ftp.ncbi.nlm.nih.gov/genbank). Genes of phage DNA polymerase, major capsid protein, RNA polymerase, head-to-tail connector protein and a terminase large subunit were extracted from the annotated genomes. Gene products in genomes annotated as “hypothetical protein CDS” were considered as known genes if their pairwise identity with known homologous was more than 50%. If there was more than one homologous gene, the sequence with the greater pairwise identity was used. Phylograms were generated based on the amino acid sequences of proteins and their concatenated alignments, using Geneious Prime and applying Clustal Omega (http://www.clustal.org/omega/) for sequence alignment with auto settings. Bacterial ribosomal proteins were extracted with RiboTree (https://github.com/philarevalo/RiboTree). Trees were constructed using the maximum likelihood (ML) method with an RAxML program [61], and with a GAMMA I BLOSUM62 protein model; the robustness of the trees was assessed by bootstrapping (1000). Heat map analysis was conducted using Gegenees [62], with accurate parameters (fragment length: 200 bp; and step size: 100 bp with the threshold set to 5%). 

### 4.7. Genome Comparison, Gene and Protein Analysis

Average nucleotide identity (ANI) was computed using the OrthoANIu tool, employing USEARCH (http://www.drive5.com/usearch/) over BLAST (https://www.ezbiocloud.net/tools/orthoaniu) [63] with default settings and Jspecies [64] (blast algorithm ANIb with 500 bp fragment length, MUMmer algorithm ANIm with default settings and tetranucleotide usage with default settings). Genome comparison was made with Easyfig [65]. The protein domain search was conducted with InterPro (http://www.ebi.ac.uk/interpro/). Protein remote homology detection, 3D structure prediction and template-based homology prediction were made using HHpred (https://toolkit.tuebingen.mpg.de/tools/hhpred) and Modeller [66]. 3D structures were visualized using UCSF ChimeraX [67]. Custom BLAST databases were mounted with the BLAST tool (https://blast.ncbi.nlm.nih.gov/). 

### 4.8. Molecular Cloning and TSP Purification

DNA sequence encoding a part of the predicted tail spike protein gp41 (AZF88104.1) of phage Arno 160 (aa residues 218–818) was PCR amplified using primers 5′-ATAGGATCCGGCACTGCAAACAATATTGC and 5′-ATACTCGAGTTACGTTCTCCTGATTCGTAT, and cloned to the plasmid pTSL using BamHI and HindIII restriction sites. Clones with inserts were identified by PCR, using the flanking primers and endonuclease hydrolysis, and verified by DNA sequencing. Recombinant protein was expressed in *E. coli* B834(DE3) by induction with 1 mM IPTG at 16 °C overnight. Cells were pelleted at 4000 g, then lysed by sonication (Virsonic, VirTis, France), resuspended in a 20 mM Tris-HCl (pH 8.0), 200 mM NaCl buffer, lysed), and then the lysate was cleared by centrifugation at 13,000 *g*. Recombinant TSP gp41 was further purified using a combination of metal-chelating and anion-exchange chromatography. Lysate was applied to a 5 mL Ni-NTA Sepharose column (GE Healthcare, Chicago, IL, USA) and proteins were eluted by a 0–200 mM imidazole step gradient in 20 mM TrisHCl (pH 8.0), 200 mM NaCl. After imidazole was removed by dialysis against 20 mM TrisHCl (pH 8.0), the 6× His-tag of the target protein was removed by TEV protease (12 h at 20 °C incubation). Final purification was carried out on a 5 mL SourceQ 15 (GE Healthcare, Chicago, IL, USA) using a linear gradient of 0–600 mM NaCl in 20 mM TrisHCl (pH 8.0). Protein concentration was determined spectrophotometrically at 280 nm, using a calculated molar extinction coefficient of 64,790 M^−1^ cm^−1^. The oligomeric state of Arno 160 gp41 was determined by gel-filtration using a calibrated Superdex 200–chromatography resin, 10 × 300–dimensions (mm) column (GE Healthcare, Chicago, IL, USA).

### 4.9. Isolation and O-Deacetylation of the O-Polysaccharide

Pca strain F160 was grown overnight in LB liquid media at 28 °C. Lipopolysaccharide was isolated from bacterial cells by phenol-water extraction [68], and contaminating nucleic acids and proteins were precipitated with aqueous 50% CCl_3_CO_2_H as described [69]. An O-polysaccharide (OPS) sample was obtained by degradation of the lipopolysaccharide (112 mg) with aqueous 2% HOAc for 1.5 h at 100 °C. A lipid precipitate was removed by centrifugation (13,000 *g*, 20 min) and the supernatant was purified by gel-permeation chromatography, using a 70 × 3.0 cm Sephadex G-50 Superfine (Amersham Biosciences, Uppsala, Sweden) column using 0.05 M pyridinium acetate buffer pH 4.5 as eluent and monitoring with a differential refractometer (Knauer, Berlin, Germany). A high-molecular mass OPS sample was obtained in a yield of 9% of the lipopolysaccharide’s weight. Total O-deacetylation was performed by incubation of OPS with 12% aqueous ammonia for 16 h at 37 °C. After evaporation of ammonia, the residue was lyophilized to yield O-deacetylated polysaccharide (DPS). 

### 4.10. Depolymerization of the Polysaccharide by the Tail Spike Protein gp41 and Isolation of the Degradation Product

The depolymerizing effect of phage Arno 160 tail spike protein gp41 was assessed by the addition of a 300 mg aliquot of the protein to the ~20 mg sample of intact (OPS) or deacetylated (DPS) polysaccharide of Pca strain F160 to the O-polysaccharide sample (20 mg), with further incubation for 2 h at room temperature. The product was isolated by gel-permeation chromatography, as described above. The reduction of viscosity was visually observed in both cases. A DPS reaction mix was applied to a column (80 × 1.6 cm) of Fractogel TSK HW-40S, and elution with 1% HOAc afforded a purified OS sample (∼12 mg). 

### 4.11. Sugar Analysis

Hydrolysis of an OPS sample (0.5 mg) was performed with 2 M CF_3_CO_2_H (120 °C, 2 h), and the monosaccharides were analyzed by gas-liquid chromatography (GLC) as alditol acetates [70] on a Maestro (Agilent 7820, Agilent, Santa Clara, CA, USA) chromatograph (Interlab, Moscow, Russia) equipped with an HP-5 column (0.32 mm × 30 m) using a temperature programme of 160 °C (1 min) to 290 °C at 7 °C/min. The absolute configuration of rhamnose was established by GLC of the acetylated (*S*)-2-octyl glycosides [32] under the same conditions as used in sugar analysis.

### 4.12. NMR Spectroscopy

Samples were deuterium-exchanged by freeze-drying from 99.9% D_2_O. NMR spectra were recorded for solutions in 99.95% D_2_O at 30 °C on a Bruker Avance II 600 MHz spectrometer (Bruker, Billerica, MA, USA) with a 5 mm broadband inverse probe head for solutions in 99.95% D_2_O at 30 °C for the O-polysaccharide, or 50 °C for the O-deacetylated polysaccharide and the oligosaccharide. Sodium 3-(trimethylsilyl) propanoate-2,2,3,3-d_4_ (δ_H_ 0, δ_C_ –1.6) was used as an internal reference for calibration. Bruker TopSpin 2.1 program was used to acquire and process the NMR data. A spin-lock time of 60 ms and mixing time of 200 ms were used in TOCSY and ROESY experiments, respectively. A two-dimensional ^1^H,^13^C HMBC experiment was recorded with a 60 ms delay for evolution of long-range couplings to optimize the spectrum for coupling constant *J*_H,C_ 8 Hz. 

## Figures and Tables

**Table 1:** ^1^H and ^13^C NMR chemical shifts (δ, ppm) of the O-deacetylated polysaccharide (DPS) from *Pectobacterium* sp. F160.

Sugar Residue	C-1	C-2	C-3	C-4	C-5	C-6
	*H-1*	*H-2*	*H-3*	*H-4*	*H-5*	*H-6* (6a,6b)
→3)-β-d-Glc*p*NAc-(1→	103.0	56.2	79.4	72.2	76.7	61.8
**A**	*4.68*	*3.75*	*3.91*	*3.69*	*3.39*	*3.88, 3.76*
→2)-α-d-Glc*p*- (1→	99.1	77.8	73.8	70.6	73.5	61.7
**B**	*5.47*	*3.65*	*3.78*	*3.49*	*3.66*	*3.82*
→2)-α-l-Rha*p*-(1→	101.3	79.1	71.1	73.55	70.6	18.0
**C**	*5.24*	*4.08*	*3.89*	*3.50*	*3.82*	*1.31*
→2)-α-l-Rha*p*-(1→	102.4	79.0	76.7	73.7	71.1	17.8
**D**	*5.17*	*4.18*	*3.95*	*3.50*	*3.70*	*1.26*
α- l-6dTalp-(1 →	103.7	70.9	67.1	73.3	68.9	17.0
**E**	*5.20*	*3.94*	*3.88*	*3.79*	*4.01*	*1.31*

^1^H NMR chemical shifts are italicized. Chemical shifts for the N-acetyl group are δ_C_ 23.8 (CH_3_) and 175.0 (CO), δ_H_ 1.95.

## Supplementary Materials

Supplementary Materials can be found at https://www.mdpi.com/1422-0067/21/9/3170/s1. Table S1. Infection range of bacteriophage Arno 160; Table S2. Putative gene functions of *Pectobacterium carotovorum* bacteriophage Arno 160; Table S3. ANIu of phage genomes calculated with OrthoANIu with 2212 *Podoviridae* Genbank genomes; Table S4. Jspecies ANIb, ANIm and regression coefficient resulting from plotting the query and target tetranucleotide signature occurrences calculated with Jspecies; Figure S1. Phylogenetic tree of 80 amino sequences of phage DNA polymerase I (RAxML,GAMMA I BLOSSUM62 protein model, with 1000 bootstrap replicates); Figure S2. Phylogenetic tree of 80 amino sequences of phage major capsid protein (RAxML,GAMMA I BLOSSUM62 protein model, with 1000 bootstrap replicates); Figure S3. Phylogenetic tree of 80 amino sequences of phage DNA-directed RNA polymerase (RAxML,GAMMA I BLOSSUM62 protein model, with 1000 bootstrap replicates); Figure S4. Phylogenetic tree of 80 amino sequences of phage head-to-tail connector protein (RAxML,GAMMA I BLOSSUM62 protein model, with 1000 bootstrap replicates); Figure S5. Phylogenetic tree of 80 amino sequences of phage large subunit of terminase (RAxML,GAMMA I BLOSSUM62 protein model, with 1000 bootstrap replicates). Figure S6. Heat-plot of the similarity matrix of 80 phage genomes made with Gegenees; Figure S7. Phylogenetic tree of 147 concatenated amino sequences of 51 ribosomal proteins (RAxML,GAMMA I BLOSSUM62 protein model, with 1000 bootstrap replicates). Eleven *Pectobacterium* strains highlighted in bold form a monophyletic clade including the strain F160 (*P. carotovorum* NCPPB 312 = ICMP 5702 = DSM 30168 type strain). *Dickeya solani* IPO 2222 was used as an outgroup.

## Abbreviations

SRP	Soft Rot *Pectobacteriaceae*
NMR	Nuclear Magnetic Resonance
Rha	Rhamnose
Tal	Talose
Glc	Glucosamine
Pca	*Pectobacterium carotovorum*
OPS	O-polysaccharide
OS	Oligosaccharide
TSP	Tail spike protein
RM	Restriction-modification
DPS	O-deacetylated polysaccharide
